# Adjuvant selenium supplementation in the form of sodium selenite in postoperative critically ill patients with severe sepsis

**DOI:** 10.1186/cc13825

**Published:** 2014-04-09

**Authors:** Yasser Sakr, Vivian PL Maia, Clesar Santos, Julia Stracke, Mohamed Zeidan, Ole Bayer, Konrad Reinhart

**Affiliations:** 1 Department of Anaesthesiology and Intensive Care, Friedrich-Schiller-University Hospital, Erlanger Allee 101, 07743 Jena, Germany; 2 Department of Anaesthesiology and Intensive Care, Theodor Bilharz Institute, El Nile Street 100, Embaba, Giza, Egypt

## Abstract

**Introduction:**

Plasma selenium (Se) concentrations are reduced in critically ill surgical patients, and lower plasma Se concentrations are associated with worse outcomes. We investigated whether adjuvant Se supplementation in the form of sodium selenite could improve outcomes in surgical patients with sepsis.

**Methods:**

In this retrospective study, all adult patients admitted to a 50-bed surgical ICU with severe sepsis between January 2004 and April 2010 were included and analysed according to whether they had received adjuvant Se supplementation, which was given at the discretion of the attending physician. When prescribed, Se was administered in the form of sodium selenite pentahydrate (Na_2_SeO_3_∙5H_2_O), in which 100 μg of Se corresponds to 333 μg of sodium selenite. A bolus of sodium selenite corresponding to 1,000 μg of Se was injected intravenously through a central venous line for 30 minutes, followed by infusion of 1,000 μg/day for 24 hours for 14 days until ICU discharge or death. We performed logistic regression analysis to investigate the impact of adjuvant Se supplementation on hospital mortality.

**Results:**

Adjuvant Se was administered to 413 (39.7%) of the 1,047 patients admitted with severe sepsis. Age and sex were similar between patients who received adjuvant Se and those who did not. Compared with patients who did not receive adjuvant Se supplementation, patients who did had higher scores on the Simplified Acute Physiology Score II, a greater prevalence of cancer upon admission to the ICU and were more commonly admitted after abdominal surgery. Compared with patients who did not receive adjuvant Se, patients who did had higher hospital mortality rates (46% versus 39.1%; *P* = 0.027), and longer median (interquartile range (IQR)) ICU stays (15 days (6 to 24) versus 11 days (4 to 24); *P* = 0.01) and hospital lengths of stay (33 days (21 to 52) versus 28 days (17 to 46); *P* = 0.001). In multivariable analysis, adjuvant Se supplementation was not independently associated with favourable outcome (odds ratio = 1.19, 95% confidence interval = 0.86 to 1.65; *P* = 0.288).

**Conclusions:**

In this retrospective analysis of a large cohort of surgical ICU patients with severe sepsis, adjuvant Se supplementation in the form of sodium selenite had no impact on in-hospital death rates after adjustment for confounders.

## Introduction

Selenium (Se) is an important trace element in human biology. It is of great importance in human health and plays a key role in thyroid function, antioxidant defence and immune function. Plasma Se levels are commonly decreased in critically ill patients for several reasons, including decreased Se intake, haemodilution by resuscitation fluids and incompletely replaced loss of biological fluids that contain large quantities of trace elements (mainly blood loss) [1,2]. In addition, Se requirements may increase while a patient has inflammatory conditions, owing to the increase in oxidative stress and production of reactive oxygen species (ROS) [2,3].

Although adjuvant Se supplementation has been shown to increase plasma selenoenzymes after 3 days [4,5], controlled clinical trials in various groups of critically ill patients [5-12] have failed to demonstrate a consistent benefit in terms of improved survival. It has also been argued that large concentrations of selenite may have oxidant properties and may result in inhibition of nuclear factor κB (NF-κB) to DNA binding [13,14] or a transient proapoptotic action on inflammatory circulating cells [15,16]. This effect has been hypothesised to attenuate the excessive inflammatory response and explain the favourable outcome in septic animals after receiving bolus doses of sodium selenite [17,18]. A meta-analysis by the Cochrane group [19] found no evidence to support use of Se supplementation for primary or secondary prevention of sepsis in critically ill patients. Authors of two other recently published meta-analyses reported a trend toward a reduction in the risk of death in patients receiving Se supplementation [20,21]. We previously reported that plasma Se concentrations were generally low in critically ill surgical patients and decreased considerably during the ICU stay in patients with organ failure, especially when lower SE concentration was attributed to infection [1]. Lower plasma Se concentrations were also associated with more tissue damage, the presence of infection or organ dysfunction/failure and increased ICU mortality [1]. Se supplementation might thus be expected to have some advantages in patients with severe sepsis. Indeed, Angstwurm *et al*. [4] found that adjuvant treatment of patients with high-dose sodium selenite might reduce mortality rates in patients with severe sepsis or septic shock, but these findings were not confirmed in a more recent study in patients with systemic inflammatory response syndrome (SIRS) and sepsis [12]. The influence of case mix on the potentially beneficial effects of Se supplementation in patients with severe sepsis is unknown, and the impact of Se supplementation on outcomes in severe sepsis patients in the postoperative setting has not previously been investigated.

The aim of our study was, therefore, to investigate the possible impact of Se supplementation on outcomes in patients with severe sepsis after major surgical procedures.

## Methods

The present study was approved by the Institutional Review Board of Friedrich Schiller University Hospital, Jena, Germany, and the need for informed consent was waived because of the retrospective, anonymous nature of the analysis. We retrospectively included all adult patients (>18 years old) admitted to our 50-bed surgical ICU with severe sepsis between January 2004 and April 2010. For patients admitted to the ICU more than once, only the first admission was considered. We excluded patients (*n* = 7) who were included in a double-blind study in which the possible influence of Se supplementation in patients with severe sepsis was being investigated.

### Data collection

Data were collected from vital sign monitors, ventilators and infusion pumps and automatically recorded using the Computer Organized Patient Record Assistant data management system (COPRA System GmbH, Sasbachwalden, Germany). This clinical information system provides staff with complete electronic documentation, order entry (for example, medications) and direct access to laboratory results. Data recorded upon admission in this study included age, sex, referring facility, primary and secondary admission diagnoses, associated comorbidities and surgical procedures preceding admission. The presence of SIRS criteria, organ failure and infection was recorded daily, together with laboratory indices of organ dysfunction/failure and markers of tissue inflammation and infection. The Simplified Acute Physiology Score II (SAPS II) [22] was calculated at the time of admission, and the Sequential Organ Failure Assessment (SOFA) score [23] was calculated daily using a special sheet by the physician in charge of the patient. A plausibility check of the automatically transmitted data was performed by the attending physician before the final scores were calculated. In sedated patients, the Glasgow Coma Scale score before the initiation of sedation was considered. Hospital mortality and hospital discharge dates were available from the electronic hospital records of all patients. In cases of clinically suspected infection, blood cultures were obtained, together with specimens from all relevant sites (bronchial aspirates, urine, catheter tip, pleural and ascitic fluids), for microbiological studies.

### Nutritional support

Patients received artificial enteral or parenteral nutritional support according to their clinical condition. Enteral formulas used in our institution during the study period included 100 μg of Se per day (Sondalis ISO; Nestlé Health Science, Liverpool, UK) by continuous perfusion through gastric or duodenal feeding tubes. Our target caloric intake was 25 kcal kg^−1^ (ideal body weight)/day, which was achieved over the course of 2 to 3 days in all patients. Additional micronutrient supplements included two vials of electrolytes and multitrace elements (INZOLEN HK; Dr F Köhler Chemie, Bensheim, Germany), each of which contained 3.1 mg of Zn, 1.8 mg of Cu and 0.99 mg of Mn, as well as one vial of multivitamins (Cernevit; Baxter Schweiz AG, Volketswil, Switzerland) containing 3.5 mg of thiamine, 11.2 IU of vitamin E and 500 mg of vitamin C. Patients fed parenterally received a multitrace element supplement (Tracitrans Plus AMP; Fresenius Kabi AG, Bad Homburg, Germany) containing 105 μg of Se in the form of sodium selenite. The current recommended dietary intake of Se in humans is between 55 and 75 μg per day [24]. The tolerable upper intake limit of Se is defined as 300 μg per day [25].

### Definitions

Severe sepsis and septic shock were diagnosed by the attending senior intensivist according to the American College of Chest Physicians/Society of Critical Care Medicine Consensus Conference criteria [26]. As routine procedure in our ICU, all patients are screened for the presence of severe sepsis by qualified research nurses, who confirm the diagnosis with the attending physicians. A list of these patients is kept for research purposes. The site of infection was documented in a special field in the patient data management system and was confirmed retrospectively by a senior attending physician (OB) for the purposes of this analysis.

Infection was defined on the basis of clinical history, clinical symptoms, physical examination and laboratory findings suggesting the presence of infection (a known or strongly suspected source of infection with positive bacterial culture for a pathogen or the presence of gross pus in a closed space) that justified administration of anti-infective therapy (excluding antimicrobial prophylaxis). Microbiologically documented infection was defined as infection supported by positive cultures of blood or body fluid from a site of suspected infection. Clinically documented infection was defined as the presence of gross pus or an abscess (confirmed anatomically and/or by imagery and/or histological evidence), but no microbiological confirmation as cultures remained sterile because of ongoing antibiotic therapy.

Organ failure was defined as a SOFA subscore >2 points for the corresponding organ. The maximum SOFA score (SOFA_max_) was defined as the highest SOFA score reached during the ICU stay and the mean SOFA score (SOFA_mean_) as the mean value throughout the ICU stay.

### Adjuvant selenium supplementation

Because of the lack of clear, universally accepted recommendations about Se supplementation during the study period, sodium selenite was prescribed in our unit at the discretion of the attending physician within 24 hours of the onset of severe sepsis. Pregnant women were not considered as candidates for Se supplementation in our ICU. When administered, Se was infused in the form of sodium selenite pentahydrate (Na_2_SeO_3_∙5H_2_O), in which 100 μg of Se corresponds to 333 μg of sodium selenite (Selenase T; Biosyn Arzneimittel, Fellbach, Germany). A bolus of sodium selenite corresponding to 1,000 μg of Se was injected intravenously through a central venous line for 30 minutes, followed by infusions of 1,000 μg/day over the course of 24 hours for 14 days until ICU discharge or death. Adjuvant Se was not repeated in the same patient during the ICU stay. Selenase T was administered in accordance with the instructions of the manufacturer. When preparing an infusion with Selenase T solution for injection as a supplement, the pH value must not fall below 7.0 and the solution must not be mixed with reducing substances (for example, vitamin C), as a precipitate of elemental Se may result. The dosage of Se used in our unit was chosen on the basis of efficacy shown in a previous pilot study [27], and it corresponds to the dosage recommended by the manufacturer for patients with sepsis. This dosage was also confirmed to be safe and effective in patients with SIRS, sepsis and septic shock in a later study [4].

### Outcome parameter

The primary outcome parameter was in-hospital death.

### Statistical analysis

The data were analysed using SPSS 17.0 for Windows software (SPSS, Chicago, IL, USA). Discrete variables are expressed as counts (percentage) and continuous variables as means ± standard deviation (SD) or median and interquartile range [IQR] unless stated otherwise. Categorical data were compared using the χ^2^ test with Yates’s correction, Fisher’s exact test or the Cochran–Armitage trend test, as appropriate. A Kolmogorov–Smirnov test was used to verify the normality of distributions of continuous variables. Continuous variables conforming to a normal distribution were compared using Student’s *t-*test; otherwise, the Mann–Whitney *U* test was applied.

To investigate the impact of Se supplementation on hospital mortality after adjusting for differences in baseline characteristics and severity of illness, we performed logistic regression analysis with in-hospital death as the dependent variable. Variables included in this analysis were age, comorbid diseases, SAPS II scores upon admission, type of surgery, primary site of infection, SOFA subscores and blood lactate levels on the day of onset of severe sepsis. Collinearity between the variables (*R*^2^ > 0.7) was checked prior to modelling, and none of the included covariates were found to be collinear. A Hosmer–Lemeshow test for goodness of fit was performed, and odds ratios (ORs) with 95% confidence intervals (CIs) were computed. All statistics were two-tailed, and *P* < 0.05 was considered to be statistically significant.

## Results

### Characteristics of the study groups

A total of 1,047 patients with severe sepsis were admitted to our surgical ICU during the study period (mean age = 67 years (±SD = 16); male = 67.4%). The characteristics of the study group are shown in Table 1. The most common surgical procedures in the whole cohort were gastrointestinal (40.8%) and cardiothoracic (31.3%). The most prevalent comorbidities upon admission to the ICU were essential hypertension (41.8%), cancer (27.7%) and insulin-dependent diabetes mellitus (25.4%). The most common sources of infection were respiratory (42.8%) and abdominal (41.3%).

**Table 1:** **Characteristics of the cohort**^**a**^

**Characteristics**	**All patients (*N*** **= 1,040)**	**No selenium (*n*** **= 627)**	**Selenium (*n*** **= 413)**	***P*-value**
Age, (yr), mean ± SD	67 ± 16	67 ± 17	67 ± 14	0.685
Male, *n* (%)	701 (67.4)	416 (66.3)	285 (69.0)	0.371
Severity scores, mean ± SD				
SAPS II, mean ± SD	48.9 ± 17.3	47.7 ± 17	50.8 ± 17.7	0.001
SOFA, mean ± SD	8.2 ± 3.5	8.2 ± 3.6	8.2 ± 3.4	0.732
Type of surgery, *n* (%)				
Gastrointestinal	424 (40.8)	204 (32.5)	220 (53.3)	<0.001
Cardiothoracic	326 (31.3)	228 (36.4)	98 (23.7)	<0.001
Trauma	48 (4.6)	35 (5.6)	13 (3.1)	0.067
Others	242 (23.3)	160 (25.5)	82 (19.9)	0.3
Comorbidities, *n* (%)				
Arterial hypertension	435 (41.8)	259 (41.3)	176 (42.6)	0.676
Diabetes mellitus	264 (25.4)	153 (24.4)	111 (26.9)	0.37
Cancer	288 (27.7)	154 (24.6)	134 (32.4)	0.005
Cirrhosis	71 (6.8)	39 (6.2)	32 (7.7)	0.339
Chronic renal failure	135 (13)	79 (12.6)	56 (13.6)	0.652
Source of sepsis, *n* (%)				
Respiratory	445 (42.8)	315 (50.2)	130 (31.5)	<0.001
Gastrointestinal	430 (41.3)	206 (32.9)	224 (54.2)	<0.001
Cardiothoracic	47 (4.5)	32 (5.1)	15 (3.6)	0.264
Wound	33 (3.2)	19 (3.0)	14 (3.4)	0.746
Urogenital	23 (2.2)	12 (1.9)	11 (2.7)	0.421
Bloodstream	11 (1.1)	6 (1.0)	5 (1.2)	0.761
Catheter	8 (0.8)	7 (1.1)	1 (0.2)	0.156
No focus identified	2 (0.2)	1 (0.2)	1 (0.2)	1.000
CNS	4 (0.4)	2 (0.3)	2 (0.5)	0.652
Others	37 (3.6)	27 (4.3)	10 (2.4)	0.108

^a^CNS: Central nervous system; SAPS II: Simplified Acute Physiology Score II; SD: Standard deviation; SOFA: Sequential Organ Failure Assessment.

### Adjuvant selenium supplementation

Se was used as an adjuvant therapy in 413 (39.7%) of the 1,047 patients. The median duration of adjuvant Se supplementation was 8 days (IQR = 4 to 12). The characteristics of the patients according to adjuvant Se supplementation are shown in Table 1. Age and sex were similar in patients who received Se and those who did not; however, patients who received adjuvant Se supplementation had higher SAPS II scores (50.8 vs. 47.7; *P* = 0.001) and a higher prevalence of cancer upon admission to the ICU (32.4% vs. 24.6%; *P* = 0.005), and they were more commonly admitted to the ICU after abdominal surgery (53.3% vs. 32.5%; *P <* 0.001) and less commonly after cardiothoracic surgery (23.7% vs. 36.4%; *P* <0.001). Abdominal sepsis (54.2% vs. 32.9%; *P* < 0.001) was more prevalent and respiratory tract infections were less prevalent (31.5% vs. 50.2%; *P* < 0.001) in the Se group than in the other patients.

The parameters of inflammation and organ function on the day of onset of severe sepsis are shown in Table 2. C-reactive protein, procalcitonin and blood lactate levels were higher in the Se group than in the other patients. Serum creatinine, platelet count, haemoglobin level and serum bilirubin level were similar between the two groups. The median mean arterial pressure (IQR) was slightly lower (59 mmHg (51 to 64) vs. 61 mmHg (55 to 67)) and the median heart rate (IQR) was higher (113 beats/min (96 to 134) vs. 108 beats/min (94 to 124)) in patients who received adjuvant Se supplementation compared to those who did not.

**Table 2:** **Physiological parameters at the onset of severe sepsis**^**a**^

**Physiological parameters**	**All patients (*N*** **= 1,040)**	**No selenium (*n*** **= 627)**	**Selenium (*n*** **= 413)**	***P-*** **value**
Leucocyte count, ×10^3^/μl	14 (8.25 to 20.3)	14.3 (9.1 to 20.2)	13.5 (6.0 to 20.7)	0.059
C-reactive protein, mg/L	101 (19 to 210)	78 (13 to 193)	146 (46 to 235)	<0.001
Procalcitonin, ng/ ml	2.7 (0.8 to 9.4)	2.3 (0.7 to 7.2)	3.7 (0.9 to 12.9)	<0.001
Blood lactate, mg/dl	2.7 (1.6 to 5.4)	2.5 (1.6 to 5.3)	2.9 (1.7 to 5.6)	0.031
Mean arterial pressure, mmHg	60 (54 to 66)	61 (55 to 67)	59 (51 to 64)	0.006
Heart rate, beats/min	110 (95 to 127)	108 (94 to 124)	113 (96 to 134)	0.003
Serum creatinine, μmol/L	102 (75 to 152)	101 (75 to 146)	108 (74 to 166)	0.257
Platelet count, ×10^3^/μl	178 (112 to 261)	176 (111 to 260)	179 (113 to 274)	0.854
Haemoglobin, mmol/L	5.5 (4.7 to 6.3)	5.5 (4.7 to 6.3)	5.5 (4.8 to 6.2)	0.757
Serum bilirubin, mg/dl	16 (10 to 27)	16 (11 to 27)	15 (10 to 28)	0.283

^a^Data presented are median (25% to 75% interquartile range).

### Morbidity and mortality

The overall ICU and hospital mortality rates were 31.3% and 41.8%, respectively (Table 3). The median ICU and hospital lengths of stay were 13 days (IQR = 5 to 24) and 30 days (IQR = 19 to 50), respectively. The ICU mortality rates were similar between the two study groups (Se vs. no Se: 33.9% vs. 29.5%; *P* = 0.135). Patients who received adjuvant Se supplementation had higher hospital mortality rates (46% vs. 39%; *P =* 0.027) and longer stays in the ICU (median = 15 days (IQR = 6 to 24) vs. 11 days (4 to 24); *P* = 0.01) and in-hospital (median = 33 days (IQR = 21 to 52) vs. 28 days (17 to 46); *P* = 0.001) than those who did not. SOFA_max_ and SOFA_mean_ scores during the ICU stay were also higher in the Se patient group than in other patients. None of the deaths of patients who received sodium selenite in our study were attributable to Se administration. In a multivariable analysis with in-hospital death as the dependent variable, adjuvant Se supplementation was not independently associated with worse outcome (OR = 1.19, 95% CI = 0.86 to 1.65; *P* = 0.288) after adjustment for age, sex, SAPS II score, type of surgery, comorbidities, focus of sepsis, SOFA subscores and blood lactate levels at the onset of severe sepsis (Table 4).

**Table 3:** **Morbidity and mortality**^**a**^

**Statistics**	**All patients (*N*** **= 1,040)**	**No selenium (*n*** **= 627)**	**Selenium (*n*** **= 413)**	***P*-value**
SOFA score, mean ± SD				
SOFA_mean_	7.9 (6.0 to 10.4)	7.6 (5.7 to 10.3)	8.3 (6.3 to 10.5)	0.002
SOFA_max_	12 (10 to 15)	12 (9 to 14)	13 (11 to 15)	<0.001
ICU mortality, *n* (%)	325 (31.3)	185 (29.5)	140 (33.9)	0.135
Hospital mortality, *n* (%)	435 (41.8)	245 (39.1)	190 (46.0)	0.027
ICU LOS (days), median (IQR)	13 (5 to 24)	11 (4 to 24)	15 (6 to 24)	0.01
Hospital LOS (days), median (IQR)	30 (19 to 50)	28 (17 to 46)	33 (21 to 52)	0.001

^a^IQR: Interquartile range; LOS: length of stay; SOFA: Sequential Organ Failure Assessment; SOFA_max_: Maximum Sequential Organ Failure Assessment score; SOFA_mean_: Mean Sequential Organ Failure Assessment score.

**Table 4:** **Summary of multivariable logistic regression analysis with in-hospital death as the dependent variable**^**a**^

**Variables**	**Univariate analysis**		**Multivariable analysis**	
	**OR (95% CI)**	***P*-value**	**OR (95% CI)**	***P*-value**
Adjuvant selenium	1.33 (1.03 to 1.71)	0.027	1.19 (0.86 to 1.65)	0.288
Age (per year)	1.03 (1.02 to 1.71)	<0.001	1.02 (1.01 to 1.04)	0.001
Sex	1.46 (0.95 to 1.65)	0.005	1.20 (0.88 to 1.65)	0.249
SAPS II score	1.09 (1.03 to 1.18)	<0.001	1.02 (1.01 to 1.03)	0.003
Type of surgery				
Gastrointestinal	2.19 (0.79 to 2.42)	<0.001	0.05 (0.24 to 1.04)	0.065
Cardiothoracic	0.68 (0.53 to 1.25)	<0.001	0.36 (0.18 to 0.75)	0.006
Trauma	1.16 (0.68 to 3.08)	0.224	0.61 (0.23 to 1.61)	0.321
Others	0.64 (0.5 to 0.85)	0.008	0.44 (0.21 to 0.93)	0.031
Comorbidities				
Arterial hypertension	0.88 (0.05 to 1.22)	0.369	1.02 (0.75 to 1.38)	0.926
Diabetes mellitus	0.93 (0.04 to 2.55)	0.621	0.77 (0.55 to 1.08)	0.129
Cancer	2.35 (1.76 to 3.06)	<0.001	2.12 (1.50 to 2.99)	<0.001
Cirrhosis	1.27 (1.07 to 2.57)	<0.001	2.63 (1.40 to 4.94)	0.037
Chronic renal failure	1.30 (1.29 to 1.75)	0.160	1.62 (1.03 to 2.55)	0.037
Source of sepsis				
Respiratory	0.96 (0.53 to 1.12)	<0.001	0.88 (0.40 to 1.91)	0.738
Gastrointestinal	1.02 (0.99 to 2.47)	<0.001	1.64 (0.73 to 3.67)	0.227
Cardiothoracic	0.99 (0.58 to 1.00)	0.090	1.01 (0.35 to 2.91)	0.985
Wound	0.99 (0.60 to 1.01)	0.177	0.59 (0.19 to 1.85)	0.362
Urogenital	0.997 (0.38 to 1.0)	0.057	0.32 (0.08 to 1.2)	0.090
Bloodstream	1.02 (1.01 to 1.16)	0.806	1.54 (0.34 to 6.86)	0.574
SOFA subscores (per point)				
SOFA resp	0.89 (0.46 to 1.08)	0.053	1.01 (0.91 to 1.13)	0.807
SOFA coag	1.26 (0.87 to 1.44)	0.001	1.18 (0.99 to 1.4)	0.063
SOFA liver	1.78 (1.44 to 3.1)	0.001	1.18 (0.98 to 1.40)	0.075
SOFA CVS	1.09 (0.90 to 1.87)	0.060	1.00 (0.89 to 1.12)	0.995
SOFA CNS	1.92 (1.02 to 3.19)	0.631	0.97 (0.86 to 1.08)	0.531
SOFA ren	1.26 (0.28 to 1.32)	<0.001	1.14 (0.96 to 1.35)	0.148
Blood lactate (mg/dl)	1.08 (0.88 to 1.09)	<0.001	1.07 (1.03 to 1.12)	0.002

^a^CI: Confidence interval; CNS: Central nervous system; coag: Coagulation; CVS: Cardiovascular system; OR: Odds ratio; ren: Renal; resp: Respiration; SAPS II: Simplified Acute Physiology Score II; SOFA: Sequential Organ Failure Assessment.

## Discussion

The main finding of our retrospective study is that adjuvant Se supplementation had no impact on hospital mortality, after adjustment for possible confounders in multivariable analysis, in a large cohort of patients with severe sepsis admitted to our ICU over a 6-year period. Patients who received adjuvant Se supplementation in our study had higher hospital mortality rates and longer ICU and hospital lengths of stay, likely because they were more severely ill as evidenced by the higher SAPS II scores upon admission to the ICU, the higher prevalence of cancer and the greater degree of tissue inflammation and hypoperfusion compared to those who did not receive adjuvant Se. Indeed, after adjustment for possible confounders, adjuvant Se supplementation was not independently associated with an increased risk of in-hospital death.

Se is an essential trace element that has antioxidant, regulatory and immune functions. Glutathione peroxidase (GPx), one of the main Se-containing enzymes, maintains membrane integrity and reduces the likelihood of propagation of oxidative damage to biomolecules through reduction of inorganic and organic peroxides [28,29]. Selenoprotein P is the most prevalent selenoprotein in plasma and has been proposed to be the most important marker of Se deficiency in sepsis [17], which supports arguments against the role of GPx as an antioxidant in plasma in the absence of glutathione in this compartment. We previously reported that plasma Se levels were lower than the standard values for healthy individuals in 92% of critically ill patients admitted to a surgical ICU [1]. Lower plasma Se levels were associated with higher degrees of tissue damage, the presence of infection and/or organ dysfunction/failure and increased ICU mortality. It has been argued that large concentrations of selenite may have oxidant properties and may result in inhibition of NF-κB to DNA binding through direct oxidation of the thiol groups of this transcription factor [13,14] or in transient proapoptotic action on inflammatory circulating cells [15,16]. This effect has been hypothesized to attenuate the excessive inflammatory response and explain the favourable outcomes in septic animals that received bolus doses of sodium selenite [17,18]. Experimental evidence of this possible mechanism of action of sodium selenite is based on studies in which researchers used higher, rather toxic, doses of sodium selenite [13-16,30]. Those studies were performed largely in the context of cancer research [13,15,16,30], not in severe sepsis. Wang *et al*. reported that the administration of a large bolus of sodium selenite (2,000 μg), rather than continuous administration, resulted in a better haemodynamic profile, fewer sepsis-induced microvascular alterations and prolonged survival time in a sheep model of peritonitis [18]. They did not observe significant differences in lipid peroxidation among the study groups at any time, however, so their results provide no information regarding a potential antioxidant or oxidant mechanism [31,32]. Interestingly, although the bolus dosage of sodium selenite in the study by Wang *et al*. was about five times higher than the dosage used in our study (0.08 mg/kg vs. 0.015 mg/kg), they reported no toxicity due to sodium selenite [18].

The antioxidant and oxidant mechanisms of action of sodium selenite both offer a plausible rationale for the use of this compound in patients with severe sepsis. In our analysis, however, we could not confirm that adjuvant Se supplementation improved outcomes in terms of in-hospital mortality in a nonselected group of patients with severe sepsis undergoing major surgical procedures. Several theories may be used to explain our findings. Although adjuvant Se supplementation may positively influence the antioxidant capacity and immune response in patients with severe sepsis [4,5,12,27,33], it may not have any direct influence on the evolution of postopeative complications and their deleterious effects on outcome. Previous researchers [4,10,12,34] who investigated the possible impact of adjuvant Se supplementation in patients with severe sepsis included mixed medical and surgical ICU patients (12.5% to 39.7% surgical), so the results of those studies cannot be extrapolated to surgical ICU patients.

The dose of adjuvant Se may also have influenced the results of our study. We used the dose employed by Angstwurm *et al*. [4], which has been shown to restore Se and GPx levels to normal values. However, the optimal dosage of Se has not been defined in a pure surgical case mix, in which Se losses may also occur during surgical procedures or via loss of body fluids through surgical drains. Higher doses of Se than those given to our patients may be required for Se supplementation to have a positive impact on outcomes in surgical ICU patients. Forceville *et al*. [10] proposed administration of high doses of Se (4,000 μg on the first day and 1,000 μg/day thereafter) as adjuvant therapy in patients with septic shock. Although those authors were not able to elucidate a beneficial effect of that dosage of Se on outcomes, probably because of the small sample size (*n* = 60), no obvious toxicity was attributable to the high Se dose. In another study involving patients with SIRS [5], a bolus loading dose of selenite delivering 2,000 μg of Se, followed by a continuous intravenous infusion of 1,600 μg/d for 10 days, was most effective at returning serum Se to physiologic levels and safely maximizing GPx activity. Likewise, in 150 patients with SIRS and/or sepsis, Valenta *et al*. [12] reported that a high dose of adjuvant Se supplementation (1,000 μg on the first day and 1,500 μg/day on days 2 to 14) increased plasma Se and GPx levels, but did not reduce mortality.

In contrast to our findings, researchers in several small studies [27,34,35] have reported beneficial effects of Se supplementation in heterogeneous groups of critically ill patients with acute pancreatitis [36] or SIRS [27,35] in terms of improved survival [36] or decreased frequency and severity of multiple organ failure [27]. Likewise, in a large, multicentre, randomized controlled trial comprising 189 patients with severe sepsis, adjuvant Se supplementation at a dosage similar to that used in our study was associated with reduced 28-day mortality (42.4% in the Se supplementation group vs. 56.7% in patients in the placebo group) [4], although the beneficial effects of Se supplementation on outcomes were observed only in the per-protocol group and not in the intention-to-treat analysis [4]. The discrepancies between the results of these studies [4,27,35,36] and ours may be attributed to differences in case mix. Our data suggest that surgical ICU patients with severe sepsis may not benefit from adjuvant Se supplementation, at least at the dosage used in our study. Future prospective, randomized studies are required to support or refute this assumption.

Selenocompounds are known to be toxic, at least in part due to their oxidant properties at high dosages [37]. The dosage of sodium selenite administered in our study has been shown in several previous studies to be safe and well-tolerated by critically ill patients [4,27]. Although the degree of organ dysfunction and/or failure during the ICU stay, as assessed by SOFA scores, was higher in patients who received sodium selenite in our study, it can be attributed to the differences in organ function already present at the time of onset of severe sepsis and before sodium selenite administration. None of the deaths of patients who received sodium selenite in our study were attributable to drug administration. This result suggests that sodium selenite, at least at the dosage applied in our study, does not have relevant acute toxic manifestations. Nonetheless, as the manifestations of sodium selenite toxicity are similar to those of sepsis-associated organ failure and may be additionally masked in sedated patients, animal studies are warranted to define safety margins regarding sodium selenite dosages. We also cannot elaborate on long-term toxicity because of the retrospective nature of the study.

To the best of our knowledge, our present study is the largest to date in which the possible impact of Se supplementation in critically ill patients with severe sepsis after major surgery has been investigated. However, our analysis has some limitations. First, the retrospective nature of the study and the absence of precise criteria upon which to establish the indications for Se supplementation during the study period may have introduced bias-by-indication because Se was given only to the more severely ill patients. Second, the multivariate analysis is limited by the included variables because of the retrospective nature of the study and the results not being adjusted for other, unmeasured variables that might have had an impact on outcomes, such as the nature and severity of surgical complications in individual patients and the septic events preceding admission to the ICU. Another possible confounding factor that was not considered in our study is the time of onset of sepsis. Also, the results of our analysis may not be extrapolated to other ICU patients, such as medical patients.

## Conclusion

In this retrospective analysis of a large cohort of surgical ICU patients with severe sepsis, adjuvant Se supplementation in the form of sodium selenite had no impact on in-hospital death rates after adjustment for possible confounders.

## Key messages

• Se is an important trace element in human biology.

• Se is of great importance in human health and plays a key role in thyroid function, antioxidant defence and immune function.

• Sodium selenite may elucidate both antioxidant and oxidant effects in severe sepsis, either or both of which may explain its potentially favourable effect on patient outcomes.

• In our large cohort of surgical ICU patients with severe sepsis, adjuvant Se supplementation in the form of sodium selenite had no impact on in-hospital death rates after adjustment for confounders.

## Abbreviations

GPx: Glutathione peroxidase; NF-κB: Nuclear factor κB; OR: Odds ratio; ROS: Reactive oxygen species; SAPS II: Simplified Acute Physiology Score II; Se: Selenium; SIRS: Systemic inflammatory response syndrome; SOFA: Sequential Ogan Failure Assessment.

## Competing interests

MZ declares he has no competing interests to disclose. YS, KR, JS, VPLM, CS and OB declare that their institution received an unrestricted grant from Biosyn GmbH to support a randomized control trial on selenium supplementation in patients with severe sepsis.

## Authors’ contributions

YS, JS and KR conceived the study. VPLM, CS, JS, MZ and OB participated in data collection. YS, VPLM and JS performed the statistical analyses. YS, VPLM, CS and MZ drafted the manuscript. KR, OB, MZ and JS revised the draft of the manuscript. All authors read and approved the final manuscript.
